# The regulation of tobacco growth under preceding crop planting: insights from soil quality, microbial communities, and metabolic profiling

**DOI:** 10.3389/fpls.2025.1530324

**Published:** 2025-02-07

**Authors:** Peiyan Zhao, Houfa Zhou, Xiaolin Liao, Leifeng Zhao, Yuanxian Zheng, Tiane Xiong, Gaorun Zhang, Sirong Jiang, Jiming Wang, Yuansheng He, Jiangtao Li, Jieying Zhu, Yongjun Zhang, Yanrun Li, Fuzhao Nian, Di Liu

**Affiliations:** ^1^ College of Tobacco Science, Yunnan Agricultural University, Kunming, Yunnan, China; ^2^ Technology and Research Center, Lincang Branch Company of Yunnan Tobacco Company, Lincang, Yunnan, China; ^3^ College of Food Science and Technology, Yunnan Agricultural University, Kunming, Yunnan, China; ^4^ College of Landscape and Horticulture, Yunnan Agricultural University, Kunming, Yunnan, China

**Keywords:** tobacco, preceding crops, soil quality, rhizosphere metabolites, soil microbial diversity

## Abstract

**Introduction:**

Specific microorganisms and metabolites in soil play key roles in regulating organismal behavior. Currently, the effects of different preceding crops on the rhizosphere soil quality of flue-cured tobacco remain unclear.

**Methods:**

Four treatments were compared in the study: fallow + tobacco (CK), maize + tobacco (T1), rapeseed + tobacco (T2), and wheat + tobacco (T3).

**Results and discussion:**

Results showed that preceding crops significantly enhanced soil nutrient levels and improved tobacco growth by altering rhizosphere metabolites and microbial community structure. Previous cultivation of maize and rapeseed significantly promoted tobacco growth, rapeseed and wheat cultivation enhanced the diversity of soil bacterial communities, and notably decreased the abundance of urea-degrading bacteria. In contrast, the preceding crop of maize reduced plant pathogenic fungi and promoted positive microbial interactions. Metabolomics analysis showed that different preceding crops altered lipids, organic acids, flavonoids, alkaloids, and terpenoids, enhancing secondary metabolite synthesis pathways in soil. Preceding crops regulated rhizosphere metabolites which potentially participated in soil carbon and nitrogen cycling, balancing soil nutrients, and improving tobacco yield. Overall, the three preceding crops altered the composition and function of metabolites and microbial community structures in rhizosphere soil, thereby increased soil nutrient concentration. Both maize and rapeseed cultivation significantly boosted tobacco growth and biomass. These findings offer new insights into the potential interactions between rhizosphere metabolites and microbial communities and strategies of comprehensively regulating tobacco growth.

## 1 Introduction

Tobacco is a significant economic crop in China’s agricultural sectors. However, in areas with limited arable land, such as Yunnan, tobacco cultivation often results in continuous cropping obstacles (Tan et al., 2021), leading to soil nutrient imbalances, reduced enzyme activity, decreased microbial diversity, and the accumulation of allelopathic substances (Ku et al., 2022), which negatively affected tobacco growth (Wang et al., 2020b). To mitigate these adverse effects, we have experimented with planting different crops during the tobacco fallow period. Diversified crop rotation has emerged as a promising approach for sustainable agriculture (Kang et al., 2020). Suitable crop diversity is crucial for enhancing soil conditions (Wang et al., 2022a). Selecting an appropriate previous crop can improve nutrient cycling, boost enzyme activity, stabilize microbial community structure and functional diversity (Trinchera et al., 2022), optimize rhizosphere metabolite composition and abundance, and enhance soil metabolic pathways (Wang et al., 2023b). Therefore, choosing suitable previous crops can effectively address the challenges of continuous cropping (Qin et al., 2017).

Corn, rapeseed, and wheat are common crops in rotation systems, contributing to soil nutrients. Maize, preceding rapeseed, enriched organic matter and available K in the rhizosphere soil (Hirzel et al., 2021). Rapeseed, as a preceding crop, significantly influences the nitrogen content of rice rhizosphere soil, thereby improving rice yields (Fang et al., 2021). Compared to other crops, wheat, with its long growth period and root residues, significantly increases soil organic matter content and urease activity (Woźniak, 2019; Chiotta et al., 2021; Neupane et al., 2021; Silva et al., 2022).

Additionally, numerous studies showed that preceding crops affect soil microbial species and abundance. For instance, maize rotation increases the relative abundance of specific soil bacteria, while rapeseed cultivation enhances microbial diversity and species symbiosis in rice rhizosphere soil (Zhang et al., 2023). Wheat cultivation supports rhizosphere-derived bacteria in soybeans, protecting them against soil-borne diseases (Yin et al., 2023). Soil microorganisms are essential for nutrient cycling, particularly carbon, nitrogen, and phosphorus (De Graaff et al., 2010), and they promote plant growth by decomposing organic matter and enhancing plant disease resistance (Adekunle et al., 2021).

The rhizosphere, a zone of complex ecological interactions among plants, soil, and microbes, is significantly influenced by root exudation (Yuan et al., 2024). Root exudation enhances plant growth by recruiting beneficial microbes, suppressing soil-borne diseases, and improving soil ecosystem multifunctionality (Seitz et al., 2022). Despite this, the combined regulatory effects of rhizosphere metabolites and soil microbes remain understudied. To address this, we propose the following hypotheses based on previous studies: (1) preceding crops influence rhizosphere metabolites via root exudates, altering the soil biochemical environment.; (2) preceding crops recruit specific microbial populations, shaping microbial community structure and function.; (3) preceding crops enhance soil nutrients and tobacco growth by modulating rhizosphere metabolites and microbial interactions. The effects of metabolites and microbial diversity on soil nutrient concentration and crop growth across various rotation systems are unclear. We aimed to determine the relative importance of edaphic factors and metabolites in shaping the microbial community. Therefore, establishing a stable and healthy soil microbiota community is crucial for plant growth, as it balances the soil environment and promotes sustainable agricultural development.

## 2 Materials and methods

### 2.1 Experimental site and soil agrochemical properties

The methods for growth regulation were conducted in a potted greenhouse at Yunnan Agricultural University (102.7479865° E, 25.1320219° N), where the average daily temperature is 17.5°C and the average daily light duration is 11.73 hours. Uncultivated red soil was utilized for the experiment. The agrochemical properties of the soil were as follows: available nitrogen was 86.4 mg/kg, available phosphorus was 22.5 mg/kg, rapid potassium was 328.57 mg/kg, and organic matter was 16.0 g/kg.

### 2.2 Experimental design and implementation

The experimental soil was sieved through a 2 mm sieve and transferred into plastic pots (42 cm × 28 cm × 40 cm), each containing 25 kg. Four treatments were established: control group (CK) with fallow + tobacco, treatment T1 with corn + tobacco, treatment T2 with rapeseed + tobacco, and treatment T3 with wheat + tobacco. Each treatment had 10 replicates. The pre-crop was sown in September of the previous year, and tobacco seedlings were transplanted in April of the following year. Each pot contained two maize or rapeseed plants, 20 wheat seeds, and 1 tobacco plant. The crop varieties used were: Yunrui 121 (corn), Youzha 50 (rapeseed), Ximai 6 (wheat), and Yunyan 87 (tobacco). Fertilization was applied using the ring-pit method (10 cm away from the plant, 10 cm deep). Fertilization was conducted twice: 60 g of compound fertilizer per pot during the previous cropping season (excluding the fallow treatment) and 60 g of compound fertilizer (N-P_2_O_5_-K_2_O = 10-10-24) per pot during the flue-cured tobacco growing season. The soil moisture was maintained at about 50%, with the same treatment applied to the fallow.

### 2.3 Soil and tobacco plant sampling

Samples were collected at the tobacco maturation stage (August of the following year), after removing the surface soil, the entire root system was extracted, and the rhizosphere soil sample was collected by gently shaking the roots. The two potting soils were combined into one sample, with five duplicate samples for each treatment. Some samples were quick frozen in liquid nitrogen for 30 min, then stored at -80°C for microbial diversity and metabolic product analysis. The remaining samples were naturally dried and sieved through a 2 mm sieve to remove impurities. Mature tobacco plants were harvested, and their roots, stems, and leaves were weighed to calculate the fresh weight of the whole plant. These parts were briefly heated at 105°C for 15 min, then dried to a constant weight at 70°C. Three replicates were collected for each treatment.

### 2.4 Soil chemical properties test

Air-dried soil samples are sieved through a 1 mm sieve, and the pH was determined using the immersion method. Organic matter was measured with potassium dichromate titration. Available nitrogen, available phosphorus, rapidly available potassium, available zinc, available boron, and available molybdenum were determined by alkali diffusion, molybdenum antimony colorimetry, flame photometry, flame atomic absorption spectroscopy, curcumin colorimetry, and potassium thiocyanate colorimetry, respectively (Tan et al., 2022). Enzyme activities (sucrase (G0302W, 540nm), urease (G0301W, 578nm), acid phosphatase (G0304W, 405nm), catalase (G0303W, 510nm)) are measured using their corresponding standard assay kits (Suzhou Grace Biotechnology Co., Ltd., China) (Borase et al., 2020), The model of the microplate reader used in the detection was KAIAO 6700FLA (Beijin, China).

### 2.5 Rhizosphere soil microbial community genomic sequencing and analysis

The E.Z.N.A.™ kit was used to extract soil microbial genomic DNA, which was then analyzed via 0.8% agarose gel electrophoresis and quantified using a NanoDrop 2000. The 16S rRNA gene’s V3-V4 region (Dai et al., 2022), corresponding to the complete bacterial community, was amplified using 338F/806R primers. Simultaneously, the fungal ITS1 gene was amplified using ITS1a and ITS1b primers (Usyk et al., 2017). After purifying the PCR products (Vazyme Biotech Co., Ltd, Nanjing, China), they were quantified using a BioTek FLx800. Paired-end sequencing was performed using the Illumina NovaSeq platform and NovaSeq 6000 SP Reagent Kit (500 cycles) (Parsortix, Shanghai, China). The QIIME 2 2019.4 platform was used for microbiome bioinformatics analysis (Ji et al., 2022), which included primer trimming, quality filtering, denoising, merging, and chimera removal.

### 2.6 Extraction and analysis of rhizosphere soil metabolites

Metabolite extraction for targeted metabolomic analysis was performed by Metware Biotechnology Co., Ltd., in Wuhan, China. Fresh soil samples (50 mg) were homogenized in 500 μl of ice-cold methanol/water (70%, v/v) for 15 minutes, then incubated and centrifuged at 4°C, 12000 rpm for 10 min. The resulting supernatant (400 μl) was transferred to a new centrifuge tube. The original tube was treated with ethyl acetate/methanol (1:3, v/v), shaken for 5 minutes, incubated on ice for 15 minutes, and then centrifuged at 4°C, 12000 rpm for 10 min, with 400 μl of the supernatant collected. The two supernatants were combined and concentrated. The dried concentrate was reconstituted with 100 µl of 70% methanol-water, sonicated for 3 min, and centrifuged at 4°C, 12000 rpm for 3 min before extraction. The soil secretions of the final extract (60 μl) underwent LC-MS/MS analysis using an Agilent 7890 high-performance liquid chromatography system (Santa Clara, CA, USA) coupled with a QTOF/MS-6545 mass spectrometer (LECO, St. Joseph, MI, USA). Data processing involved the use of ProteoWizard software and the XCMS program for data conversion, peak extraction, alignment, retention time correction, and filtering to obtain metabolic identification information. Subsequently, the R package ropls (Version 1.6.2) was employed for data analysis.

### 2.7 Data processing and analysis

The sequence data analysis primarily utilized QIIME2 and R packages (v3.2.0). A network analysis of the top 50 abundant bacterial and fungal communities was conducted using microbial ASVs to examine interspecies relationships. Gephi visualization (v 0.10.1) was employed to explore symbiotic patterns in soil microbial communities based on strong (*p* > 0.6) and significant (*p* < 0.01) correlations. Node sizes corresponded to connectivity with other nodes, and coloration reflected genus classification. Additionally, Spearman analysis of soil properties, bacterial and fungal abundances, and α-diversity indices was performed using SPSS 26.0 software. KEGG pathway enrichment analysis was applied to analyze the differential metabolites. Pearson correlation analysis was used to investigate relationships among rhizosphere soil nutrients, metabolites, and microbial diversity.

## 3 Results

### 3.1 Impact of three different preceding crops on tobacco biomass accumulation

**Figure 1:**
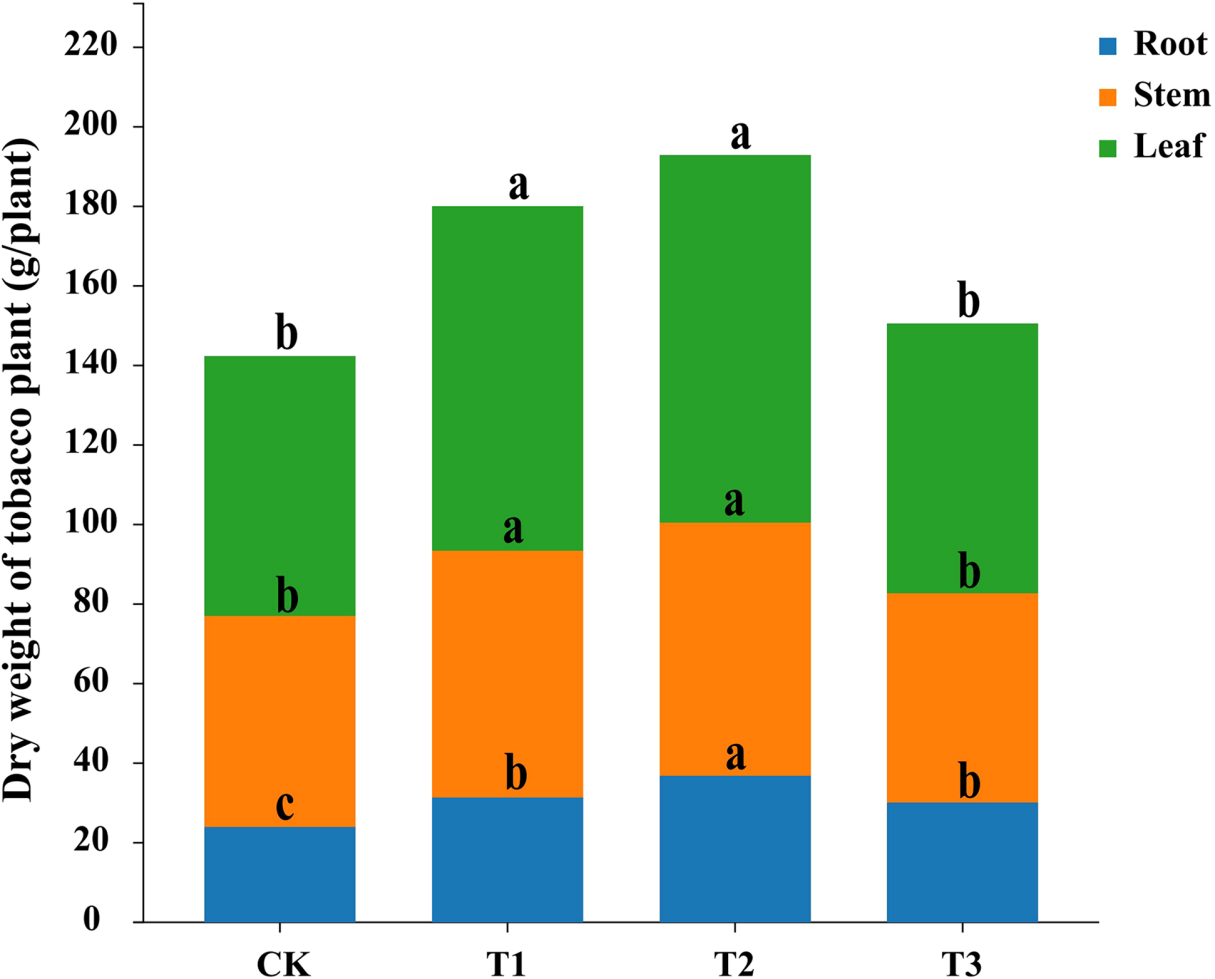
Impact of three different preceding crops on tobacco biomass accumulation in different organ. fallow + tobacco (CK), maize + tobacco (T1), rapeseed + tobacco (T2), and wheat + tobacco (T3), different letters indicated significant differences between treatments at *p* < 0.05 level, the same below.

Compared to fallow (CK), preceding crops maize (T1), rapeseed (T2), and wheat (T3) all enhanced tobacco growth. Former crop planting of maize and rapeseed significantly increased the dry weights of tobacco roots, stems, and leaves (*p* < 0.05), the dry weight of tobacco was not significantly affected by former crop planting of wheat, but it significantly increased the dry weight of tobacco root (*p* < 0.05). Among the preceding crops, rapeseed had the greatest impact on tobacco biomass accumulation (**Figure 1**).

### 3.2 Effect of three different preceding crops on agrochemical properties and enzyme activities of tobacco rhizosphere soil

**Figure 2:**
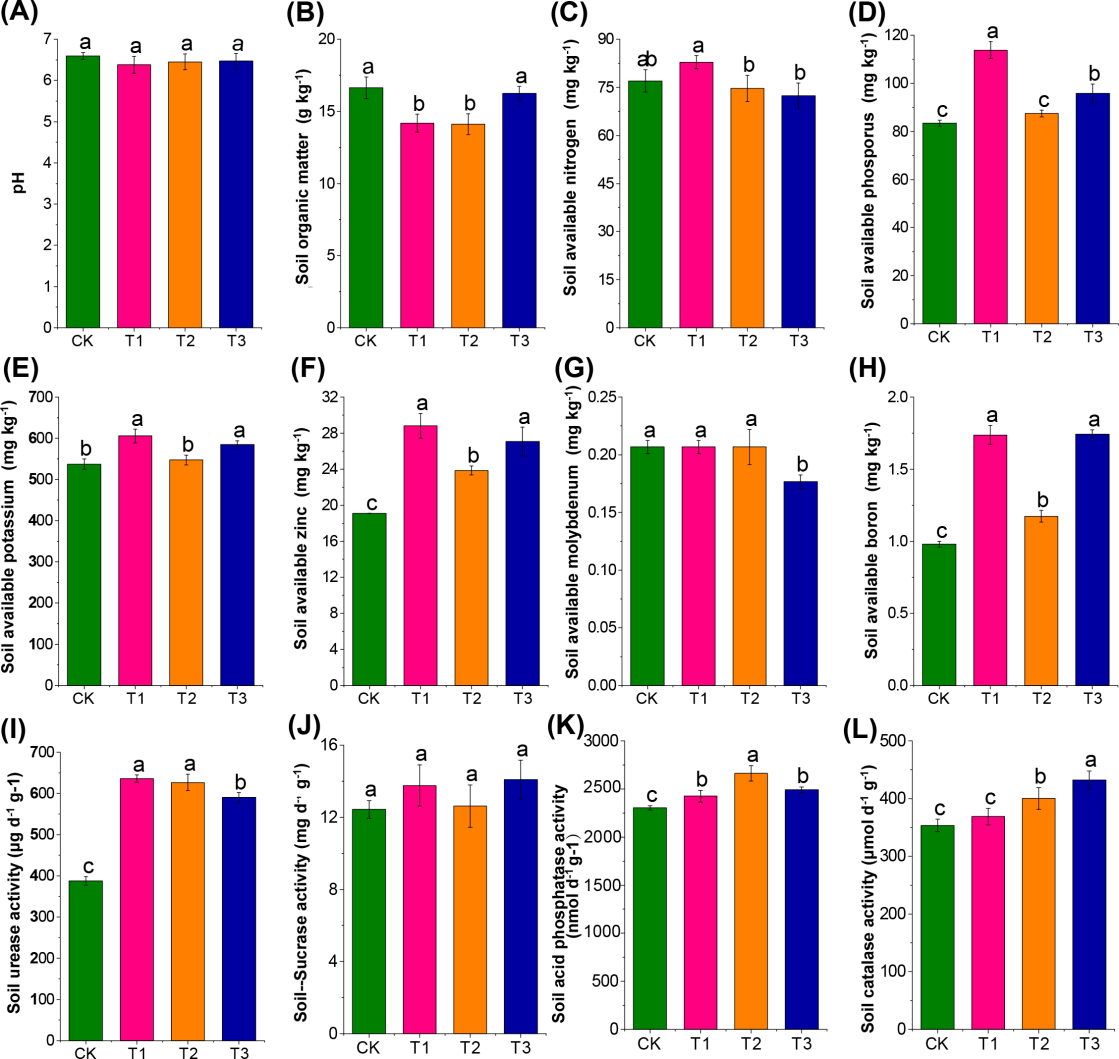
Effect of three different preceding crops on soil agrochemical properties and enzyme activities in the tobacco rhizosphere. **(A)** pH, **(B)** Soil organic matter, **(C)** Soil available nitrogen, **(D)** Soil available phosphorus, **(E)** Soil available potassium, **(F)** Soil available zinc, **(G)** Soil available molybdenum, **(H)** Soil available boron, **(I)** Soil urease activity, **(J)** Soil sucrase activity, **(K)** Soil acid phosphatase activity, **(L)** Soil catalase activity, with different letters indicated significant differences between treatments at *p* < 0.05 level.

Previous crops significantly influenced soil nutrient concentration and enzyme activities compared to fallow conditions (*p* < 0.05). Both T1 and T3 treatments significantly increased available phosphorus and potassium content compared to fallow (**Figures 2A–E**). All three treatments demonstrated notably higher effective zinc and boron content, urease, and acid phosphatase activities than the fallow group (CK). Additionally, the T2 and T3 groups exhibited significantly higher catalase activities than CK (**Figures 2F–L**). These results indicate that cultivation of maize and rapeseed promoted phosphorus and potassium accumulation in the soil. A decrease in soil organic matter was noted after cultivating maize and canola. Notably, all three preceding crops positively improved the availability of zinc and boron and the activities of carbon and nitrogen-related enzymes.

### 3.3 Influence of three preceding crops on soil microbial diversity in the tobacco rhizosphere

**Figure 3:**
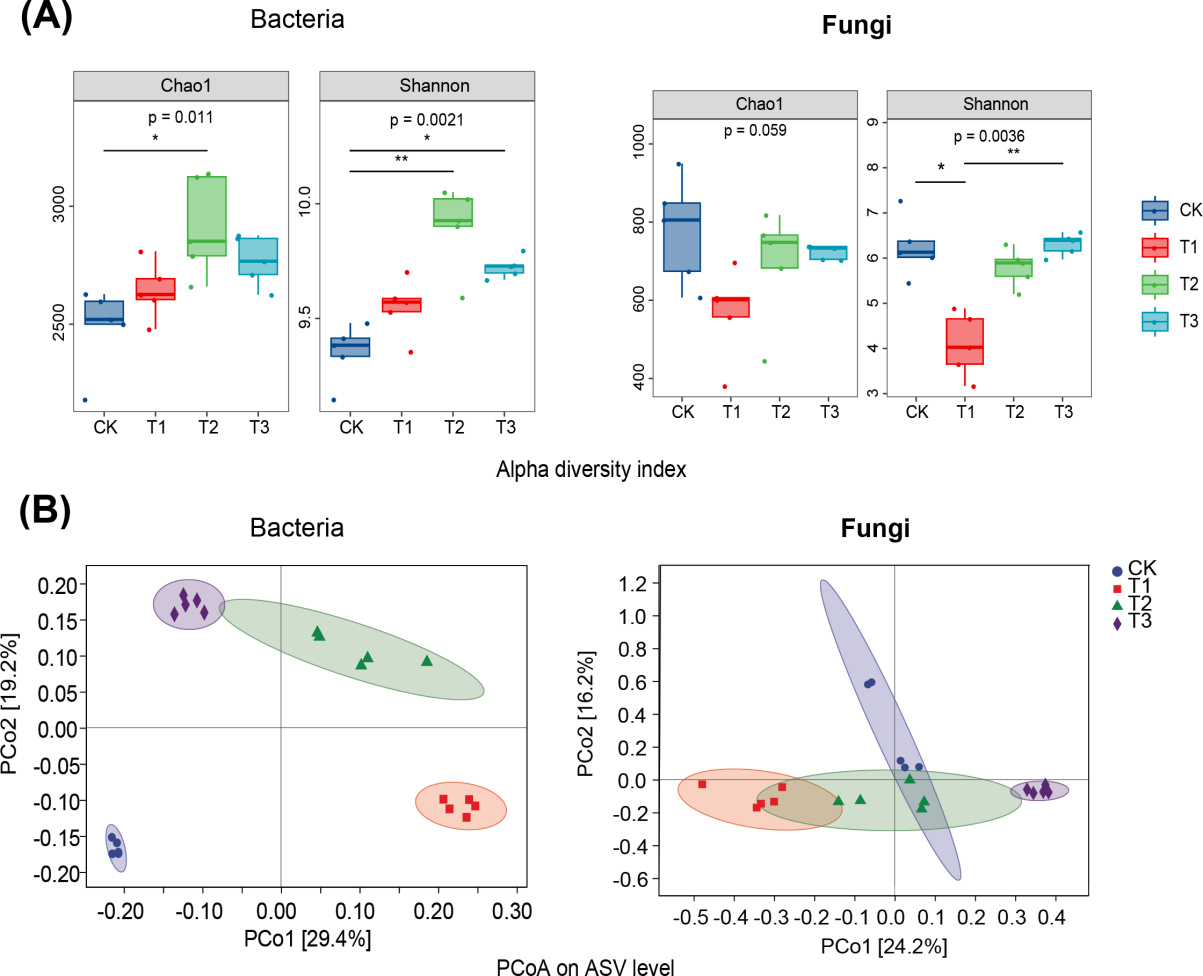
Effect of three preceding crops on soil microbial diversity of Chao1 index, Shannon index and Principal Coordinate Analysis (PCoA) in the tobacco rhizosphere. **(A)** α diversity, **(B)** Principal Coordinate Analysis.

Rapeseed cultivation significantly enhanced the Chao1 and Shannon indices of soil bacteria compared to fallow (*p* < 0.05, *p* < 0.01), with wheat also significantly increasing the Shannon index (*p* < 0.05). These results indicate that preceding crops notably increased the abundance of soil bacteria in tobacco rhizosphere soil. PCoA analysis showed distinct bacterial communities in tobacco rhizosphere soil following different preceding crops and fallow. In contrast, fungal communities exhibited greater clustering across treatments (**Figures 3A, B**), with maize as a preceding crop differing significantly from fallow. This suggests that preceding crops significantly impact bacterial and fungal communities in tobacco rhizosphere soil, with maize preceding notably affecting fungal communities.

### 3.4 Impact of three preceding crops on the composition of soil microbial communities in the tobacco rhizosphere soil

**Figure 4:**
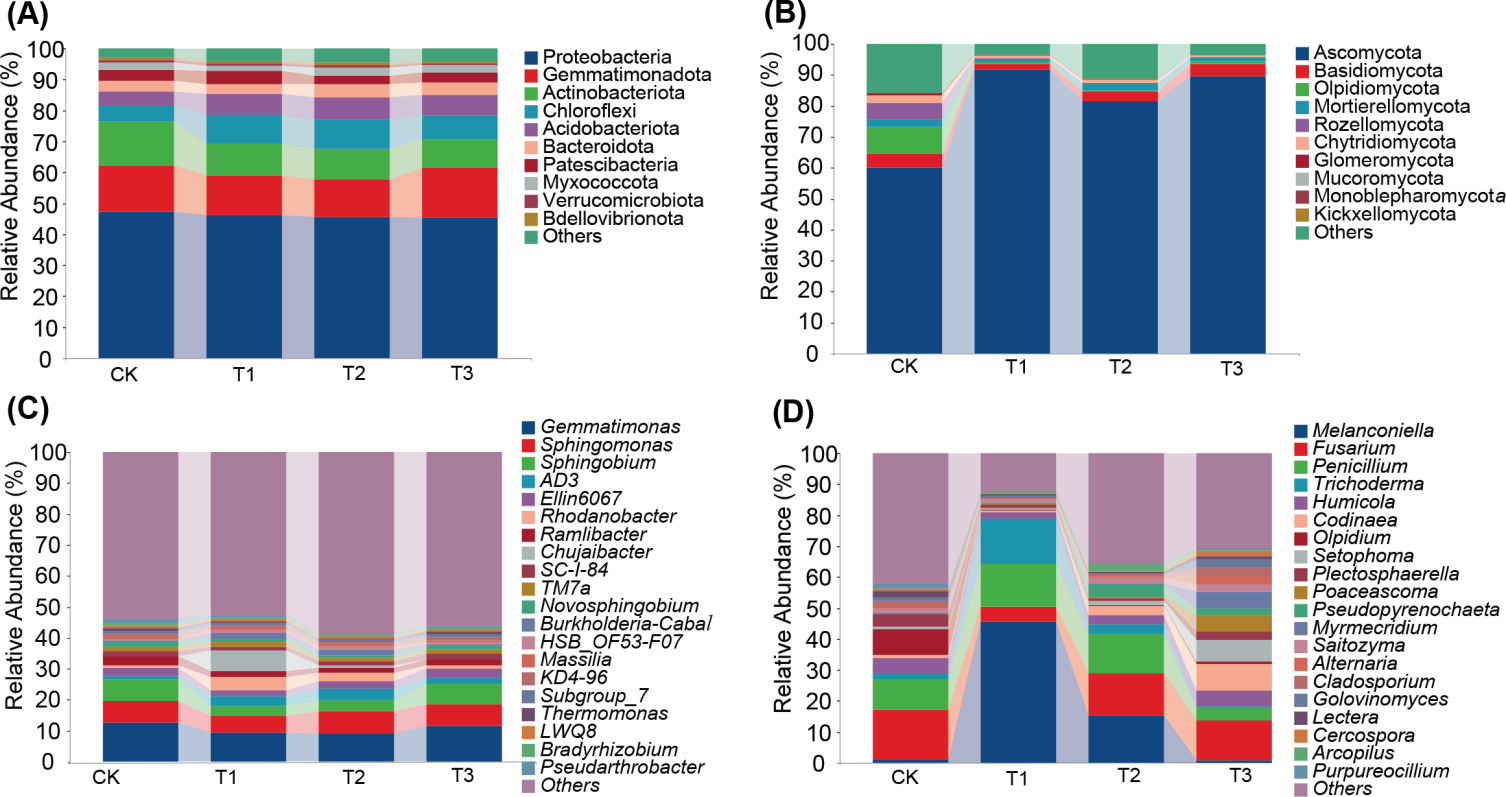
Impact of three preceding crops on the soil microbial community composition at the phyla and genus levels in the tobacco rhizosphere. relative abundance of **(A)** bacteria at phyla level, **(B)** fungi at phyla level, **(C)** bacteria at genus level, **(D)** fungi at genus level.

The predominant bacterial phyla in tobacco rhizosphere soil across all treatment groups were Proteobacteria, Gemmatimonadota and Actinobacteria, along with eight others, comprising over 90% of the total abundance. The T2 treatment group showed the highest relative abundance of Acidobacteriota. Notably, Chloroflexi and Patescibacteria were more abundant in T1 treatment compared to other treatments. Similarly, the dominant fungal phyla including Ascomycota, Basidiomycota, Olpidiomycota, Mortierellomycota, and Chytridiomycota constituted over 80% of the total abundance. The T1 treatment demonstrated a significantly higher relative abundance of Ascomycota compared to other treatments (**Figures 4A, B**).

At the genus level, the top 20 relatively abundant bacterial genera in tobacco rhizosphere soil included *Gemmatimonas*, *Sphingomonas*, *Sphingobium*, *AD3*, and *Ellin6067*. Notably, *Rhodanobacter* and *Chujaibacter* showed the highest relative abundance in T1 treatment. Among fungal genera, *Melanconiella*, *Fusarium*, *Penicillium*, *Trichoderma*, and *Humicola* were predominant. *Melanconiella* was dominant in T1 and T2, *Trichoderma* was most abundant in T1, and *Codinaea* exhibited the highest relative abundance in T3. The variation of fungal communities among different treatment in rhizosphere soil exceeded bacterial communities (**Figures 4C, D**). These findings suggest that maize as a preceding crop had a more significant impact on the relative abundance of soil bacteria, whereas all three preceding crops substantially influenced the relative abundance of soil fungi.

### 3.5 Impact of three preceding crops on microbial functions and co-occurrence networks in tobacco rhizosphere soil

**Figure 5:**
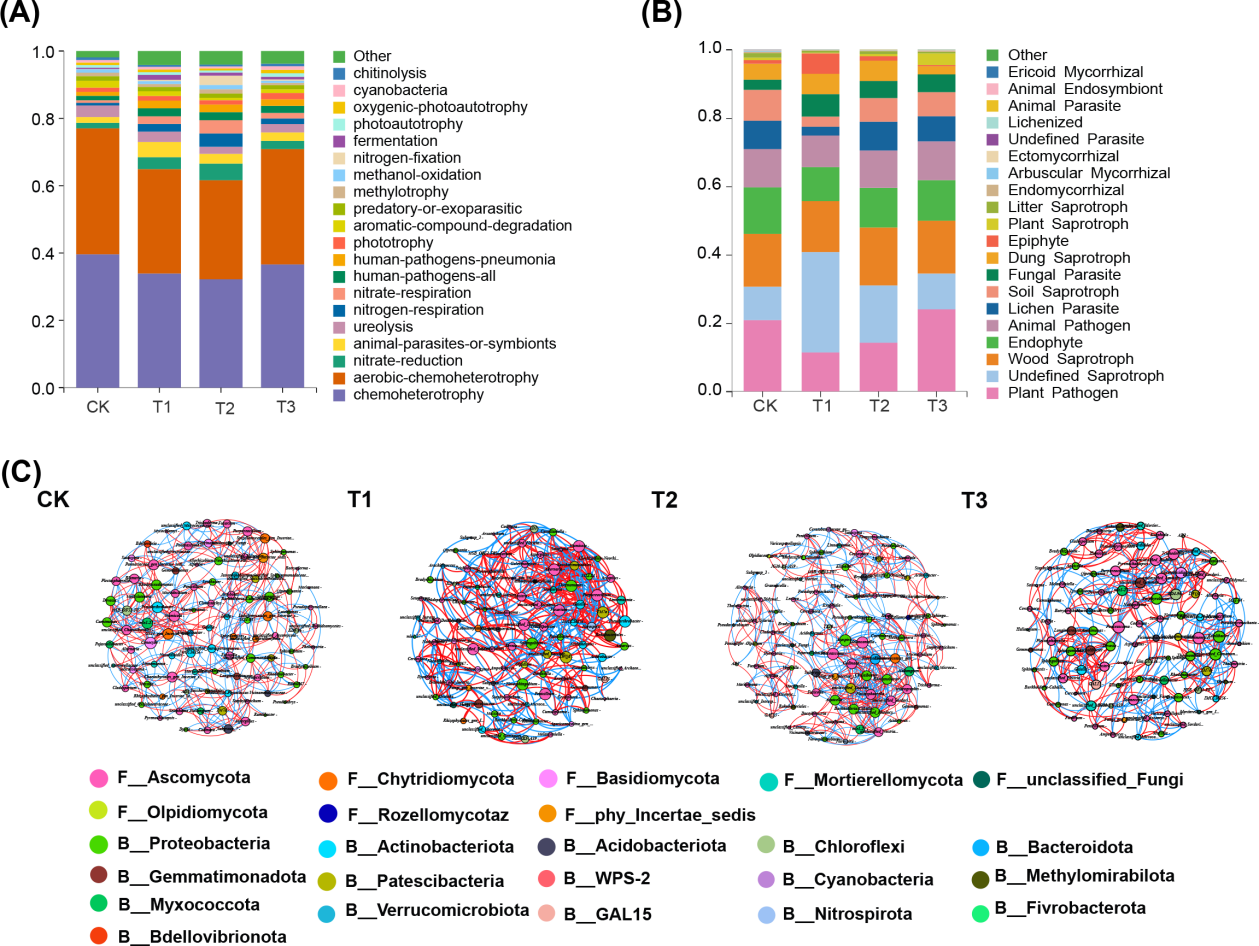
Impact of three preceding crops of microbial functions prediction and co-occurrence networks of bacteria and fungi in the tobacco rhizosphere soil. **(A)** bacterial function prediction based on the FATROTAX database, **(B)** fungal function prediction based on the FUNGuild database, **(C)** bacteria and fungi co-occurrence network in the tobacco rhizosphere soil. Lines between different genus indicates a significantly (*p* < 0.01) strong positive association (red, Spearman’s ρ > 0.6) or negative association (blue, Spearman’s ρ < -0.6). The size of each node is proportional to the number of connections, with same color nodes belonging to the same phyla.

The FATROTAX database was utilized to predict soil bacteria functions, revealing an assessment of 62 categories (**Figure 5A**). After cultivating three distinct preceding crops, a significant decrease in the relative abundance of chemolithotrophic bacteria was observed, along with an increase in symbiotic animal functional bacteria compared to fallow groups. Notably, the presence of urea-degrading bacteria in tobacco soil decreased significantly with rapeseed and wheat as predecessors, while nitrate-reducing bacteria increased significantly with maize and rapeseed as predecessors (*p* < 0.001). For soil fungal communities, functional predictions during various preceding crop maturity stages were made using the FUNGuild database (**Figure 5B**), which categorizes nutritional modes into pathotrophs, saprotrophs, and symbiotrophs. Maize as a predecessor led to a significant reduction in lichen parasites, plant pathogens, and soil saprotroph fungi compared to fallow conditions (*p* < 0.05), and a significant increase in fungal parasites and undefined saprotroph fungi (*p* < 0.01). However, there was also an increase in fungal parasites in treatments using rapeseed and wheat as precursors, but no significant difference was observed.

The rhizosphere soil of tobacco was analyzed to identify the top 50 bacterial and fungal genera, investigating co-occurrence networks under different predecessor conditions (**Figure 5C**; **Table 1**). Microbial community structure and co-occurrence patterns varied, with rapeseed and wheat showing more complex networks compared to other planting patterns. Key nodes in the co-occurrence networks included *Conocybe*, *Humicola*, *Pseudarthrobacter*, *TM7a*, *Thermomonas*, *Sphingobium*, *Purpureocilliu*, *Poaceascamom*, *mle1-27*, *Lectera*, and *Gaiella*. The proportions of positive and negative correlations differed among networks: maize had 55.37% positive and 44.63% negative, rapeseed had 51.13% positive and 48.87% negative, wheat had 50.80% positive and 49.2% negative, and fallow had 52.03% positive and 47.97% negative. Mutualistic relationships prevailed over competitive ones, with maize showing the strongest mutualistic interaction, followed by rapeseed, while wheat exhibited the weakest mutualistic interaction.

**Table 1:** Topological characteristics of co-occurrence networks of bacteria and fungi in the rhizosphere soil of flue-cured tobacco under three preceding crops.

Index	Modularity (MD)	Average clustering coefficient	Average path length	Network diameter	Grath density	Average degree (AD)	Positive	Negative	Nodes	Edges
CK	0.581	0.542	3.694	8	0.083	8.041	205	189	98	394
T1	0.535	0.575	3.592	8	0.106	10.634	304	245	100	549
T2	0.589	0.585	4.134	11	0.098	9.74	249	238	100	487
T3	0.703	0.615	4.067	9	0.088	8.74	222	215	100	437

### 3.6 Impact of three preceding crops on differential metabolite analysis in the tobacco rhizosphere soil

**Figure 6:**
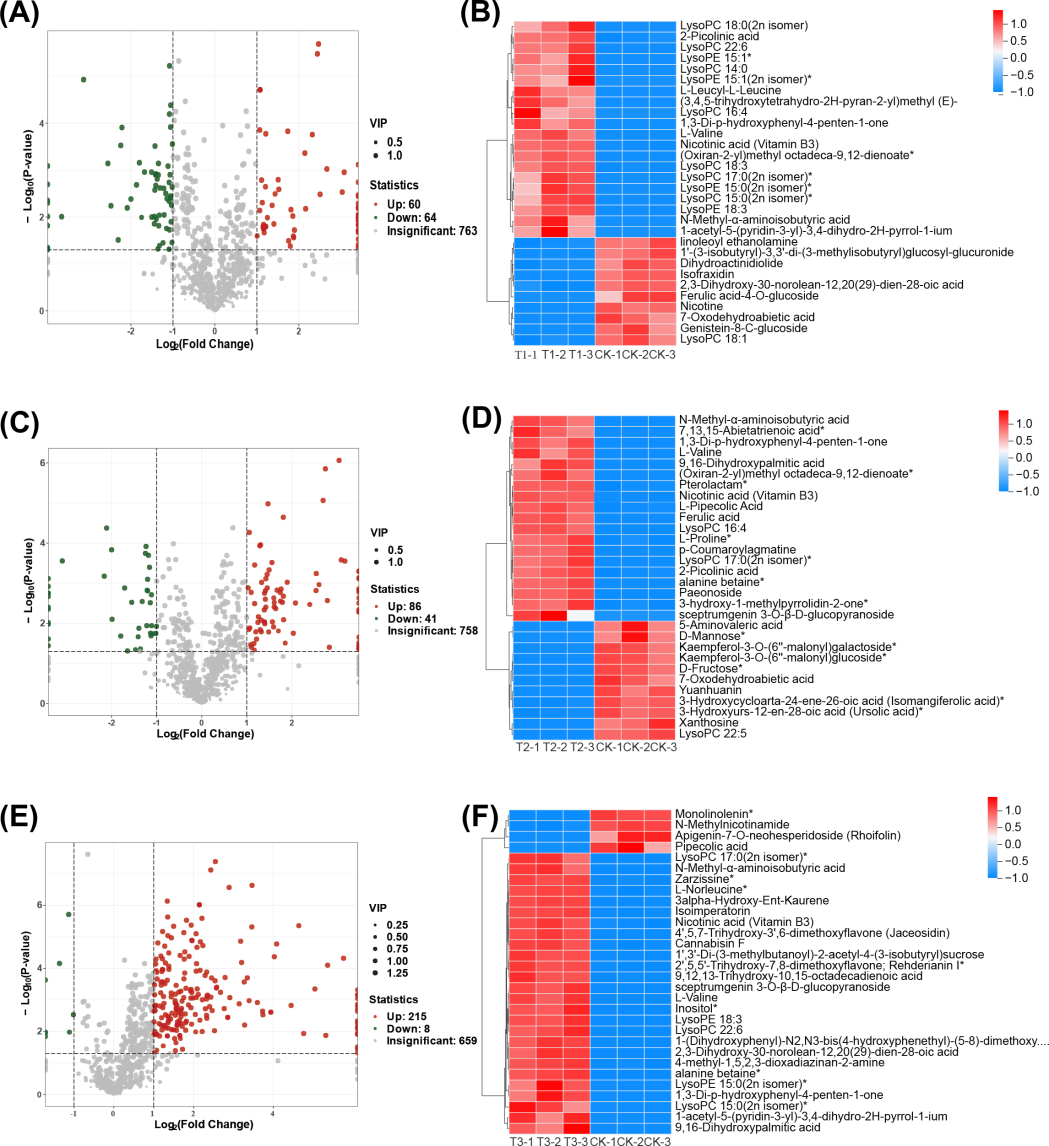
Differential metabolite analysis. **(A, C, E)** are the volcano plots of up and down-regulated metabolites of T1 vs CK, T2 vs CK, and T3 vs CK, respectively. **(B, D, F)** are differential metabolite cluster analysis charts (Top30) of T1 vs CK, T2 vs CK and T3 vs CK, with the color indicating the relative metabolite expression in the group of samples.

Volcano plots were employed to classify differential metabolites, compare with CK, identifying 124, 127, and 223 differential metabolites in T1, T2, and T3 treatments, respectively. These metabolites included lipids, flavonoids, alkaloids, amino acids and derivatives, terpenoids, and other compounds. Compared to fallow conditions (CK), T1, T2, and T3 treatments exhibited 60, 86, and 215 upregulated metabolites, and 64, 41, and 8 downregulated ones (**Figures 6A, C, E**; **Supplementary Table S1**). Using the OPLS-DA model, VIP values were calculated, compared to fallow conditions, cluster analysis (Top30) showed significant upregulation and downregulation of 20 and 10 metabolites in T1, 19 and 11 in T2, and 26 and 4 in T3 treatment, respectively, (CK) (**Figures 6B, D, F**). These results suggest that both rapeseed and wheat previous crops enhance the upregulation of differential metabolites in soil, with wheat demonstrating the most pronounced upregulation trend.

### 3.7 Differential metabolite enrichment in KEGG pathways and key metabolic pathways

**Figure 7:**
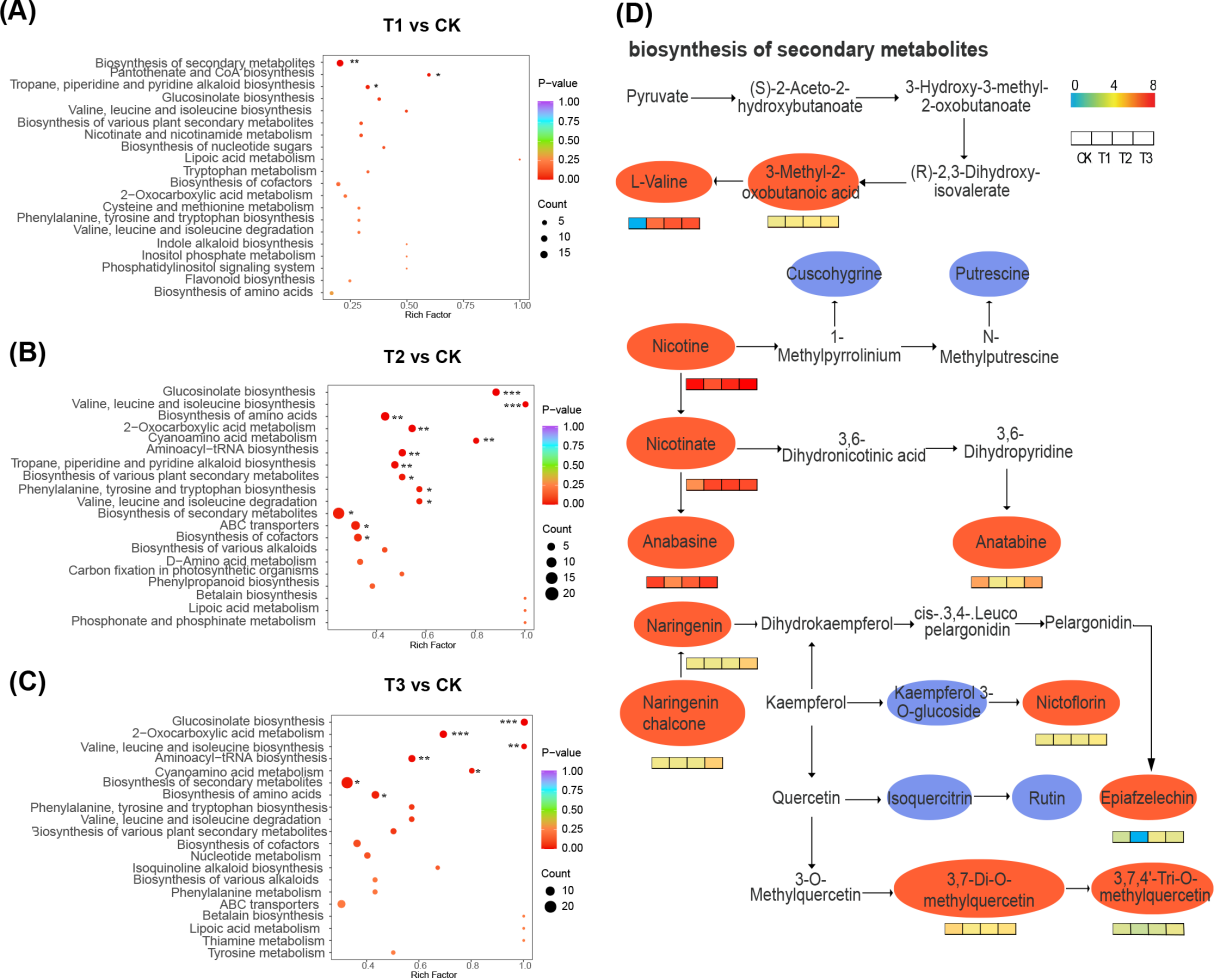
Differential metabolite enrichment in KEGG pathways and key metabolic pathways. **(A-C)** are the differential metabolite enrichment in KEGG pathways of T1 vs CK, T2 vs CK, and T3 vs CK, respectively. **(D)** is the highly enriched pathway in the key metabolic pathways (secondary metabolic pathway), with the ellipse representing the metabolite in the pathway. Red indicates a significant difference, while blue indicates no significant difference.

The fallow group (CK) differed significantly from T1 treatment, which exhibited enrichment in biosynthesis of secondary metabolites, pantothenate and CoA biosynthesis, terpenoid, pyridine, and piperidine alkaloids (*p* < 0.05) (**Figure 7A**). In contrast, T2 treatment demonstrated significant enrichment in gluconasturtiin, valine, leucine, and isoleucine amino acids, 2-oxocarboxylic acid metabolism, and cyanogenic amino acid metabolism, among others (*p* ≤ 0.05) (**Figure 7B**). T3 treatment showed significant enrichment in gluconasturtiin, 2-oxocarboxylic acid, valine, leucine, isoleucine, aminoacyl-tRNA, cyanogenic amino acid metabolism, and other pathways (*p* ≤ 0.05) (**Figure 7C**). Furthermore, compared to the fallow planting pattern, the rhizosphere differential metabolites were significantly enriched in secondary metabolite biosynthesis after planting three different crops. Therefore, the degree of enrichment in secondary metabolite biosynthesis under the four treatments was presented. Soil under both rapeseed and wheat as preceding crops showed a significant increase in the abundance of L-valine, 3-methyl-2-oxo-butanoic acid, nicotine, and nicotinic acid (**Figure 7D**).

### 3.8 Conjoint analysis

**Figure 8:**
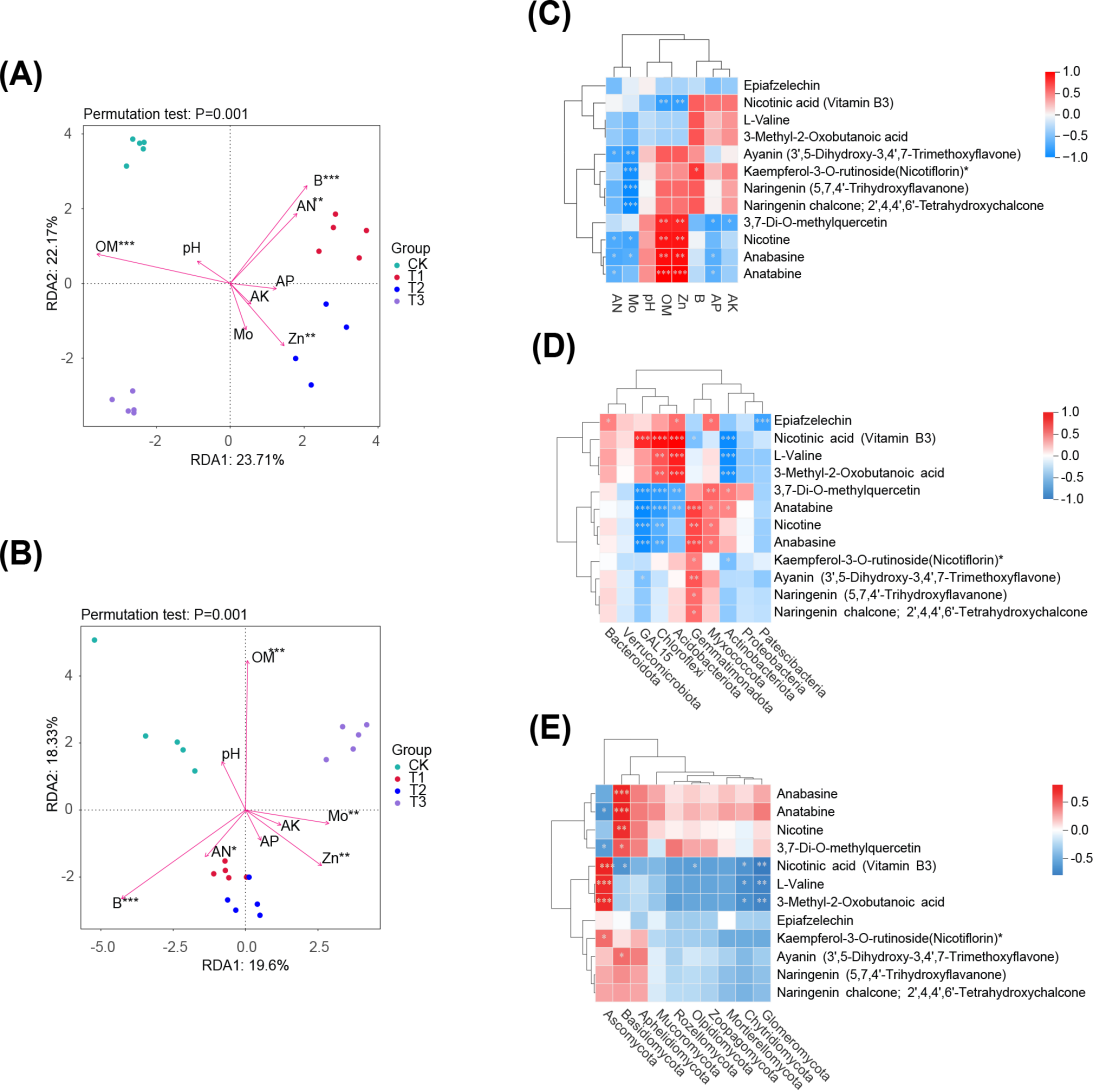
Conjoint analysis. **(A, B)** are the redundancy analysis between soil environmental variables and microbial community structure of bacteria and fungi, respectively. **(C)** indicates the correlation analysis between differential metabolites of key metabolic pathways and soil environmental indexes, **(D)** and **(E)** are the correlations between soil microbial community structure (bacteria and fungi) and differential metabolites in key metabolic pathways, respectively. Significance level: *** *p* < 0.001, ** 0.001 ≤ *p* < 0.01, * 0.01 ≤ *p* < 0.05.

The relationship between soil environmental variables and microbial community was assessed using RDA analysis. In the bacterial community structure of tobacco soil, RDA1 and RDA2 explained 23.71% and 22.17% of the variance, respectively. Organic matter (OM), available nitrogen (AN), zinc (Zn), and boron (B) had significant effects on bacterial community composition (*p* < 0.01) (**Figure 8A**). Similarly, in the fungal community structure of tobacco soil, RDA1 and RDA2 accounted for 19.6% and 18.33% of the variation, respectively. Environmental factors including OM, Zn, B, molybdenum (Mo), and AN significantly influenced fungal community composition (*p* ≤ 0.05) (**Figure 8B**).

A correlation analysis was performed between soil environmental factors and the peak area of differential metabolites in key metabolic pathways (**Figure 8C**). This analysis showed that certain key metabolites such as Nicotine, Anabasine, significantly affected the levels of available nitrogen, effective molybdenum and zinc, and organic matter, the metabolites demonstrated a positive correlation with effective zinc and organic matter content, and a negative correlation with available nitrogen and effective molybdenum content.

To investigate the relationship between differential metabolites in rhizosphere soil and microbial communities, a correlation analysis was conducted focusing on the top 10 phyla by relative abundance in the microbial community and the peak area of differential metabolites in key metabolic pathways. Significant correlations were identified between the bacterial phyla GAL15, Chloroflexi, Acidobacteriota, Gemmatimonadota, Myxococcota, and Actinobacteria with more than five key differential metabolites, including nicotinic acid and 3,7-Di-O-methylquercetin (**Figure 8D**). Similarly, the fungal phyla Ascomycota and Basidiomycota also showed significant correlations with over five key differential metabolites, such as 3,7-Di-O-methylquercetin, nicotinic acid, and anatabine (**Figure 8E**). These findings indicate that microbial phyla are influenced by changes of key metabolic substance and pathways of various preceding crops.

## 4 Discussion

This study demonstrated that all three preceding crops increased tobacco biomass compared to fallow conditions. Another study noted that different preceding crops can enhance plant growth by improving nutrient availability until maturity (Zhang et al., 2022), resulting in superior tobacco growth compared to fallow conditions. Specifically, tobacco cultivated after rapeseed showed the highest biomass, highlighting the substantial influence of different preceding crops on planting patterns (Chichongue et al., 2022). Soil nutrients directly influence crop growth in agriculture (Wulanningtyas et al., 2021; Vaziritabar et al., 2024). This study found that soil with maize and wheat as preceding crops exhibited significantly higher levels of available phosphorus and potassium compared to fallow conditions and rapeseed predecessors. The increase in available phosphorus may be attributed to excessive phosphorus fertilizer application (Yan et al., 2023), while the decrease in available potassium in soils with rapeseed as a predecessor due to the high utilization rate of rapeseed for potassium absorption (Gu et al., 2024). Zinc acts as a catalyst for protein synthesis and activates numerous enzymes, while boron contributes to tobacco carbon metabolism and transport (Vera-Maldonado et al., 2024; Singh et al., 2024). Different predecessor treatments significantly boosted soil available zinc and boron levels, partially fulfilling the demands of both previous crops and tobacco for trace elements (Brodowska et al., 2022; Palmer et al., 2023). Predecessor planting could enhance soil enzyme activity, modify nutrient cycling, and influence matter and energy metabolism (Wang et al., 2022b). Soil urease and acid phosphatase activities significantly increased under all three predecessor treatments compared to fallow conditions, with soil catalase activity particularly rising under rapeseed and wheat predecessor treatments. These changes improve soil fertility and nitrogen nutrition, mitigate the harmful effects of hydrogen peroxide, and enhance available phosphorus accumulation (Borase et al., 2020), thus benefiting the growth of subsequent crops.

Soil microbes play a critical role for sustainable agriculture by influencing soil fertility through following processes, such as organic carbon decomposition, humus formation, and soil nutrient transformation cycles (Xu et al., 2024). Modularization of microbial interactions is crucial for maintaining stability and resilience of microbial communities, influenced by resource allocation, habitat heterogeneity, phylogenetic status, and ecological niche overlap (Qiao et al., 2024). A recent study has identified a modular structure in rhizosphere soil microbial networks, which varies with different predecessor crops (Liu et al., 2020). This finding underscores that different crops influence the rhizosphere soil’s microbial diversity (Kowalchuk et al., 2002; Chaparro et al., 2014; Li et al., 2014). Additionally, predecessor crops can enhance soil bacterial abundance and diversity, thereby promoting crop growth (Xi et al., 2021).

Redundancy analysis demonstrated a significant correlation between soil physicochemical properties and soil microbial composition. Different predecessor crops primarily affect the bacterial phyla as Acidobacteriota, Chloroflexi, and Patescibacteria. Acidobacteriota decompose soil plant litter and utilize carbohydrates from root exudates (De Chaves et al., 2019). After rapeseed cultivation, Acidobacteriota abundance increased, facilitating carbohydrate transformation in the rhizosphere. Chloroflexi are crucial for soil organic matter metabolism and community establishment (Nabi et al., 2022). In contrast, Patescibacteria, which have minimal known functions in soil environments, require further study (Wang et al., 2023a). The abundances of Chloroflexi increased after maize cultivation, enhancing plant photosynthesis and carbon metabolism. Ascomycota, which often comprise over 90% of fungal species (Wu et al., 2020), also significantly increased after maize cultivation. Ascomycota are key in degrading lignin-rich organic matter and releasing nutrients for plant growth. Root exudates strongly influence the diversity and types of active fungal populations (Jin et al., 2022). Different predecessor crops alter the rhizosphere microbial community in tobacco, potentially affecting plant-microbe interactions and nutrient cycling, which benefits tobacco growth.

Metabolite analysis of plants and rhizospheric organisms has revealed that specific metabolites detected in the rhizosphere soil of crops are primarily plant root exudates (Shi et al., 2023). The biosynthesis of secondary metabolites in the soil is significantly influenced by previous crops such as maize, rapeseed, and wheat, with plants playing a more important role than microorganisms (Bi et al., 2021; Li et al., 2022). Variations in rhizospheric metabolites primarily come from exudates of preceding crops, impacting plants growth, soil characteristics, and microbial communities (Tiziani et al., 2022). Importantly, this study indicated that maize and wheat cultivation resulted in increased available phosphorus concentration in soil compared to planted fallow, however, acid phosphatase activity was highest in soils where the previous crop was rape, the phosphorus efficiency of the three preceding crop treatments was improved, possibly due to the release of organic acids and enzymes in root secretions, enhancing the solubility of phosphorus compounds and thereby increasing their bioavailability (Chai and Schachtman, 2021). Alkaloids, nitrogen-containing compounds, are closely associated with nitrogen (Wang et al., 2023b), with three key alkaloid metabolites nicotine, anabasine, anatabine showing a negative correlation with mineral nitrogen levels in soil.

The study established a interdependent relationship between the microbial community and metabolites, with microbial community stability and diversity significantly influencing metabolite types and abundance. Conversely, plant root exudate metabolites shape the rhizosphere soil microbiota and drive plant-soil feedbacks on plant growth (Adamczyk et al., 2021). However, the impact of rhizosphere metabolites from varying predecessor crops on microbial community interactions in tobacco cultivation remains unclear. RDA and correlation analyses showed that soil environmental factors (e.g., OM, AN, Zn, B) significantly influence microbial community structure, while metabolites (e.g., nicotine, 3,7-Di-O-methylquercetin) are closely related to soil nutrient content. These metabolites may regulate microbial community composition, improving the rhizosphere environment and promoting tobacco growth. The correlation analysis revealed that Actinobacteria, the primary bacterial phylum, was influenced by rhizosphere metabolites. Actinobacteria often utilize flavonoids as substrates to produce diverse secondary metabolites (Wang et al., 2020a). Notably, six key flavonoid compounds in the metabolic pathways and L-valine were negatively correlated with Actinobacteria. Previous studies indicated that elevated amino acid levels, such as L-valine, may inhibit the growth of some bacteria (Moe, 2013). This inhibitory effect may indirectly improve the rhizosphere environment of tobacco by reducing the competitiveness of pathogens, thereby contributing to the enhancement of tobacco growth performance (Wu et al., 2024). In addition, high concentrations of amino acids may serve as a nutrient source for certain non-pathogenic microbes, promoting their proliferation and indirectly suppressing pathogens (Gong et al., 2023). This mechanism may provide a novel indirect protection strategy for tobacco growth. Notably, L-valine exhibited the most significant increase and variation under all three predecessor crops compared to fallow conditions, suggesting its potential role as a marker metabolite affecting soil bacterial diversity. In summary, the study demonstrated that different predecessor crops influence the composition of metabolic substances and the function of microbial communities in the rhizosphere through root exudates. These variations also affected soil nutrient concentrations and regulate tobacco growth. However, the study’s limitations include the specific soil and management practices used for potting, necessitating further validation through field experiments (Yuan et al., 2024).

## 5 Conclusion

This study examines how different preceding crops affect rhizosphere soil nutrients, microbial diversity, and metabolite profiles of tobacco, and their combined impact on tobacco growth. The results indicate that preceding canola, wheat, and maize significantly increased available phosphorus, potassium, boron, and zinc in the rhizosphere soil. Both canola and wheat enhanced soil bacterial diversity while reducing nitrogen-transforming bacteria. Conversely, maize reduced fungal pathogens and positively influenced microbial populations. Notably, wheat cultivation had the greatest impact on rhizosphere metabolites, increasing the abundance of most differential metabolites. The changes in metabolites of tobacco following maize and canola cultivation promoted tobacco growth, whereas no effect was observed after wheat cultivation, indicating that shifts in metabolite profiles may not always benefit tobacco growth. This study confirms that changes in key metabolites influence microbial communities, shaping the rhizosphere microenvironment and enhancing tobacco growth. Overall, this research demonstrates that rhizosphere metabolites influenced by different preceding crops regulate subsequent crop growth and soil improvement, offering insights for sustainable ecological tobacco cultivation.

## Data Availability

Microbial data associated with this article can be found in the online version. Raw sequencing data were deposited in the NCBI Sequence Read Archive (SRA, Bacteria: https://www.ncbi.nlm.nih.gov/sra/PRJNA1105736; Fungi: https:/www.ncbi.nlm.nih.gov/sra/PRJNA1105742) with Accession No. PRJNA1105736, No. PRJNA1105742. All other raw data was transferred to Mendeley data as Microsoft Excel files (https://data.mendeley.com/drafts/2c9vjc6s46).
