# Supplementary figures and images for: The regulation of tobacco growth under preceding crop planting: insights from soil quality, microbial communities, and metabolic profiling
Source: Front Plant Sci. 2025 Feb 7;16:1530324. doi: 10.3389/fpls.2025.1530324 (PMC11842363; doi:10.3389/fpls.2025.1530324)

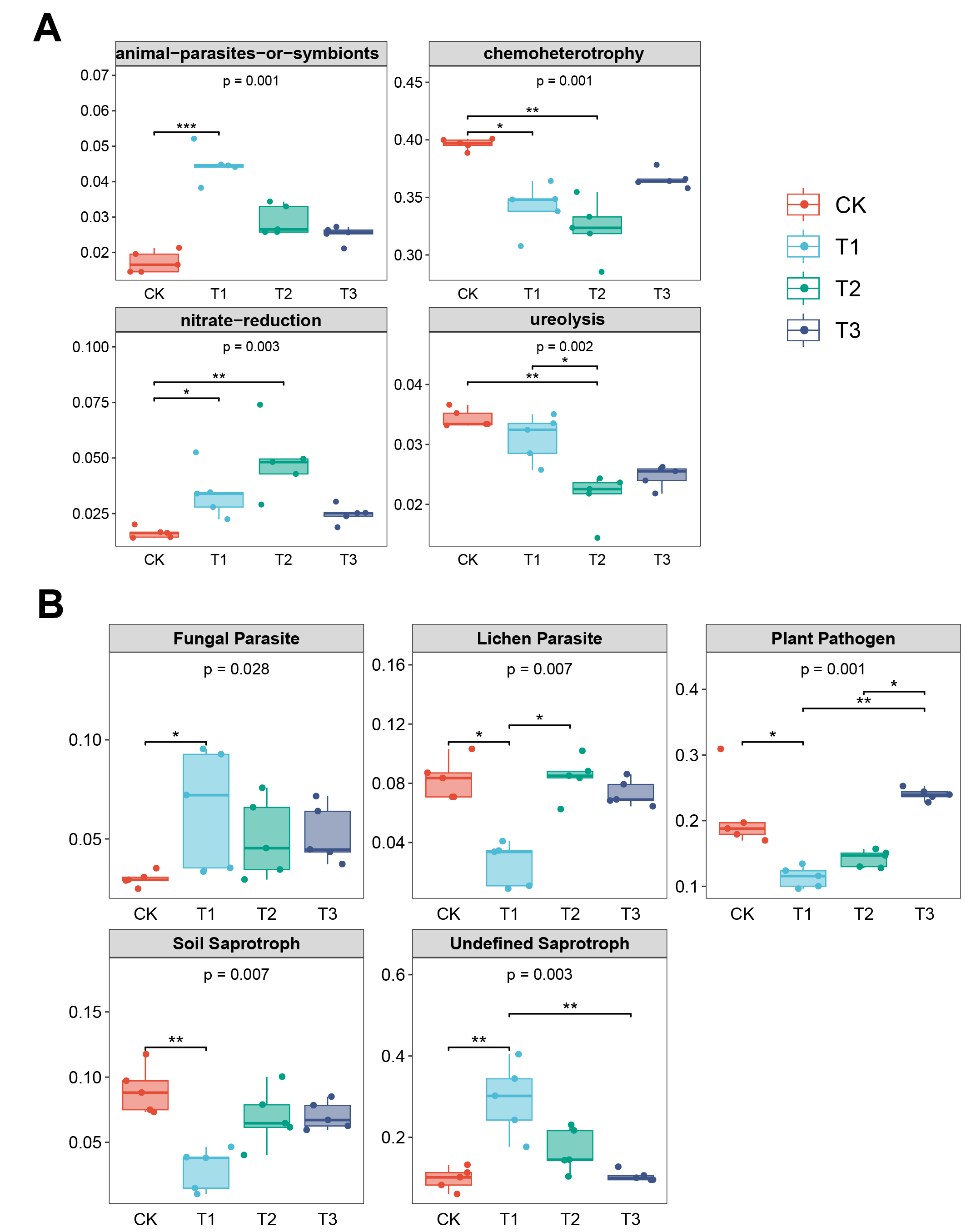

Supplement: Supplementary file 1 [file Image1.tif]
