# Supplementary material for: The regulation of tobacco growth under preceding crop planting: insights from soil quality, microbial communities, and metabolic profiling
Source: Front Plant Sci. 2025 Feb 7;16:1530324. doi: 10.3389/fpls.2025.1530324 (PMC11842363; doi:10.3389/fpls.2025.1530324)
Supplement: Supplementary file 2 [file Table1.docx]

Table S1 Differential metabolites

|  | Metabolite | Metab ID | Type | VIP | P-value | FDR |
| --- | --- | --- | --- | --- | --- | --- |
| T1vsCK | Genistein-8-C-glucoside | Lmhp009368 | down | 1.40 | 0.01 | 0.04 |
|  | Isofraxidin | Walp012785 | down | 1.40 | 0.00 | 0.02 |
|  | (3,4,5-trihydroxytetrahydro-2H-pyran-2-yl)methyl (E)-3-(4-hydroxy-3-methoxyphenyl)acrylate | Lmhp008801 | up | 1.40 | 0.01 | 0.05 |
|  | L-Valine | Lmhp008440 | up | 1.40 | 0.00 | 0.02 |
|  | L-Leucyl-L-Leucine | ZbYn008624 | up | 1.40 | 0.01 | 0.05 |
|  | 1,3-Di-p-hydroxyphenyl-4-penten-1-one | Lmhp010515 | up | 1.40 | 0.01 | 0.04 |
|  | LysoPC 22:6 | Lmhp009129 | up | 1.40 | 0.00 | 0.03 |
|  | (Oxiran-2-yl)methyl octadeca-9,12-dienoate* | Lmhp008273 | up | 1.40 | 0.00 | 0.02 |
|  | Nicotine | pmd0130 | down | 1.40 | 0.00 | 0.02 |
|  | Nicotinic acid (Vitamin B3) | Lmhp008885 | up | 1.40 | 0.00 | 0.00 |
|  | LysoPE 18:3 | pmd0136 | up | 1.40 | 0.00 | 0.03 |
|  | LysoPE 15:1* | pmb0854 | up | 1.40 | 0.01 | 0.05 |
|  | 1'-(3-isobutyryl)-3,3'-di-(3-methylisobutyryl)glucosyl-glucuronide | pmp001281 | down | 1.40 | 0.00 | 0.03 |
|  | N-Methyl-α-aminoisobutyric acid | Lmhp007595 | up | 1.40 | 0.01 | 0.06 |
|  | LysoPC 17:0(2n isomer)* | Lcsp013328 | up | 1.40 | 0.01 | 0.06 |
|  | LysoPC 15:0(2n isomer)* | MWS2430 | up | 1.40 | 0.01 | 0.06 |
|  | 7-Oxodehydroabietic acid | Lmhp009682 | down | 1.40 | 0.00 | 0.04 |
|  | LysoPE 15:1(2n isomer)* | pmp001251 | up | 1.40 | 0.02 | 0.08 |
|  | LysoPC 14:0 | Zbfn007626 | up | 1.40 | 0.01 | 0.06 |
|  | LysoPE 15:0(2n isomer)* | Lmhp008688 | up | 1.40 | 0.01 | 0.07 |
|  | 1-acetyl-5-(pyridin-3-yl)-3,4-dihydro-2H-pyrrol-1-ium | pmb0865 | up | 1.40 | 0.02 | 0.09 |
|  | LysoPC 18:0(2n isomer) | Lmyn007883 | up | 1.40 | 0.01 | 0.06 |
|  | LysoPC 18:3 | Lmhp010908 | up | 1.40 | 0.00 | 0.02 |
|  | LysoPC 18:1 | Waln010192 | down | 1.40 | 0.00 | 0.02 |
|  | Dihydroactinidiolide | Walp013004 | down | 1.40 | 0.00 | 0.03 |
|  | LysoPC 16:4 | Lmyn006011 | up | 1.40 | 0.02 | 0.08 |
|  | 2-Picolinic acid | Waln010743 | up | 1.40 | 0.00 | 0.01 |
|  | Ferulic acid-4-O-glucoside | ZbYn008751 | down | 1.40 | 0.02 | 0.07 |
|  | linoleoyl ethanolamine | Lmhp011388 | down | 1.40 | 0.00 | 0.02 |
|  | 2,3-Dihydroxy-30-norolean-12,20(29)-dien-28-oic acid | Lmhp009890 | down | 1.40 | 0.00 | 0.00 |
| T2vsCK | LysoPC 22:5 | Lmhp010099 | down | 1.42 | 0.00 | 0.02 |
|  | Xanthosine | mws0668 | down | 1.42 | 0.00 | 0.03 |
|  | Ferulic acid | mws0014 | up | 1.42 | 0.00 | 0.02 |
|  | N-Methyl-α-aminoisobutyric acid | MWS5209 | up | 1.42 | 0.00 | 0.02 |
|  | alanine betaine* | Hahp000801 | up | 1.42 | 0.00 | 0.02 |
|  | LysoPC 16:4 | Lmhp007595 | up | 1.42 | 0.00 | 0.01 |
|  | D-Fructose* | mws1164 | down | 1.42 | 0.00 | 0.02 |
|  | Nicotinic acid (Vitamin B3) | pme0490 | up | 1.42 | 0.00 | 0.00 |
|  | Yuanhuanin | Cmyp002064 | down | 1.42 | 0.00 | 0.02 |
|  | L-Valine | mws0256 | up | 1.42 | 0.01 | 0.04 |
|  | p-Coumaroylagmatine | pmb0508 | up | 1.42 | 0.00 | 0.02 |
|  | Paeonoside | Zamp003418 | up | 1.42 | 0.00 | 0.01 |
|  | LysoPC 17:0(2n isomer)* | Lmhp010515 | up | 1.42 | 0.00 | 0.02 |
|  | 1,3-Di-p-hydroxyphenyl-4-penten-1-one | Latp006960 | up | 1.42 | 0.00 | 0.02 |
|  | Kaempferol-3-O-(6''-malonyl)galactoside* | Lmdp004892 | down | 1.42 | 0.00 | 0.02 |
|  | Kaempferol-3-O-(6''-malonyl)glucoside* | Lmmp003817 | down | 1.42 | 0.00 | 0.02 |
|  | D-Mannose* | pmf0138 | down | 1.42 | 0.01 | 0.06 |
|  | 9,16-Dihydroxypalmitic acid | Lmyn007883 | up | 1.42 | 0.00 | 0.03 |
|  | (Oxiran-2-yl)methyl octadeca-9,12-dienoate* | Walp012785 | up | 1.42 | 0.00 | 0.03 |
|  | 3-Hydroxyurs-12-en-28-oic acid (Ursolic acid)* | mws4053 | down | 1.42 | 0.00 | 0.02 |
|  | 5-Aminovaleric acid | pme0120 | down | 1.42 | 0.01 | 0.05 |
|  | Pterolactam* | MWStz040 | up | 1.42 | 0.00 | 0.00 |
|  | 7,13,15-Abietatrienoic acid* | Zaxn005821 | up | 1.42 | 0.01 | 0.04 |
|  | L-Pipecolic Acid | MWS0811 | up | 1.42 | 0.00 | 0.01 |
|  | 3-Hydroxycycloarta-24-ene-26-oic acid (Isomangiferolic acid)* | Lmmn008971 | down | 1.42 | 0.00 | 0.01 |
|  | 7-Oxodehydroabietic acid | Wbmn009020 | down | 1.42 | 0.00 | 0.03 |
|  | L-Proline* | pme0006 | up | 1.42 | 0.00 | 0.02 |
|  | 2-Picolinic acid | pme1216 | up | 1.42 | 0.00 | 0.01 |
|  | 3-hydroxy-1-methylpyrrolidin-2-one* | Yacp000453 | up | 1.42 | 0.00 | 0.02 |
|  | sceptrumgenin 3-O-β-D-glucopyranoside | Lahp007183 | up | 1.42 | 0.04 | 0.13 |
| T3vsCK | alanine betaine* | Hahp000801 | up | 1.34 | 0.00 | 0.00 |
|  | sceptrumgenin 3-O-β-D-glucopyranoside | Lahp007183 | up | 1.34 | 0.00 | 0.01 |
|  | 4',5,7-Trihydroxy-3',6-dimethoxyflavone (Jaceosidin) | pmp000004 | up | 1.34 | 0.00 | 0.00 |
|  | N-Methyl-α-aminoisobutyric acid | MWS5209 | up | 1.34 | 0.00 | 0.01 |
|  | L-Valine | mws0256 | up | 1.34 | 0.00 | 0.01 |
|  | Monolinolenin* | Lmsp010763 | down | 1.34 | 0.00 | 0.00 |
|  | Inositol* | Hmln000297 | up | 1.34 | 0.00 | 0.01 |
|  | LysoPE 18:3 | Lmhp008801 | up | 1.34 | 0.00 | 0.01 |
|  | LysoPC 17:0(2n isomer)* | Lmhp010515 | up | 1.34 | 0.00 | 0.01 |
|  | LysoPC 22:6 | Lmhp009368 | up | 1.34 | 0.00 | 0.01 |
|  | 1,3-Di-p-hydroxyphenyl-4-penten-1-one | Latp006960 | up | 1.34 | 0.01 | 0.02 |
|  | Zarzissine* | Lmxp000939 | up | 1.34 | 0.00 | 0.00 |
|  | 1',3'-Di-(3-methylbutanoyl)-2-acetyl-4-(3-isobutyryl)sucrose | ZbYn008582 | up | 1.34 | 0.00 | 0.00 |
|  | 2,3-Dihydroxy-30-norolean-12,20(29)-dien-28-oic acid | Zjmp102714 | up | 1.34 | 0.00 | 0.01 |
|  | 3alpha-Hydroxy-Ent-Kaurene | Sazp011702 | up | 1.34 | 0.00 | 0.00 |
|  | 1-(Dihydroxyphenyl)-N2,N3-bis(4-hydroxyphenethyl)-(5-8)-dimethoxy-1,2dihydronaphthalene-2,3-dicarboxamide | pmp001187 | up | 1.34 | 0.00 | 0.00 |
|  | LysoPE 15:0(2n isomer)* | Lmhp008885 | up | 1.34 | 0.01 | 0.02 |
|  | 1-acetyl-5-(pyridin-3-yl)-3,4-dihydro-2H-pyrrol-1-ium | Zbqp002537 | up | 1.34 | 0.01 | 0.02 |
|  | Nicotinic acid (Vitamin B3) | pme0490 | up | 1.34 | 0.00 | 0.00 |
|  | LysoPC 15:0(2n isomer)* | Lmhp009129 | up | 1.34 | 0.01 | 0.03 |
|  | Cannabisin F | pmp001253 | up | 1.34 | 0.00 | 0.00 |
|  | N-Methylnicotinamide | pme0489 | down | 1.34 | 0.00 | 0.00 |
|  | Isoimperatorin | pmf0526 | up | 1.34 | 0.00 | 0.00 |
|  | L-Norleucine* | mws1587 | up | 1.34 | 0.00 | 0.00 |
|  | Pipecolic acid | Ysjp000315 | down | 1.34 | 0.01 | 0.04 |
|  | 4-methyl-1,5,2,3-dioxadiazinan-2-amine | Zmmp002106 | up | 1.34 | 0.00 | 0.00 |
|  | 2',5,5'-Trihydroxy-7,8-dimethoxyflavone; Rehderianin I* | Hmhp005160 | up | 1.34 | 0.00 | 0.00 |
|  | 9,12,13-Trihydroxy-10,15-octadecadienoic acid | pmn001691 | up | 1.34 | 0.00 | 0.01 |
|  | Apigenin-7-O-neohesperidoside (Rhoifolin) | MWSmce498 | down | 1.34 | 0.01 | 0.03 |
|  | 9,16-Dihydroxypalmitic acid | Lmyn007883 | up | 1.34 | 0.01 | 0.03 |
